# Supplementary material for: Are carbon emissions trading and green financial instruments synergistic? -Comprehensive quantitative research based on content analysis
Source: PLoS One. 2024 Mar 7;19(3):e0298601. doi: 10.1371/journal.pone.0298601 (PMC10919601; doi:10.1371/journal.pone.0298601)
Supplement: S1 Data — (ZIP) [file pone.0298601.s001.zip › Data/地级市Z_F工作报告.zip!/║╙─╧/┬σ╤⌠╩╨/└┤╫╘╣1⁄2╓┌║┼í╛┬φ┐╦╩2╛▌═°í┐_2017.docx]

各位代表：

　　现在，我代表市人民政府，向大会报告工作，请予审议，并请各位政协委员和其他列席人士提出意见。

　　一、2016年工作回顾

　　2016年是“十三五”的开局之年。一年来，全市上下紧紧围绕“9+2”工作布局和“四高一强一率先”奋斗目标，积极推进供给侧结构性改革，统筹稳增长、促改革、调结构、强基础、惠民生、防风险各项工作，经济社会呈现良好发展态势。

　　——综合实力迈上新台阶。预计全年实现地区生产总值3740亿元，同比增长8.8%，增速保持全省第一方阵；一般公共预算收入302.7亿元，增长9.1%；固定资产投资4085亿元，增长15.5%；社会消费品零售总额1800亿元，增长12.5%。

　　——结构调整取得新成效。全市高技术产业增加值同比增长16.8%，六大高成长性制造业增加值同比增长13.3%，分别高于规模以上工业增速7.9和4.4个百分点；第三产业增加值占GDP的比重达44.3%，对经济增长贡献率达56.6%，产业结构正在由“二三一”向“三二一”渐进。

　　——创新发展步入新阶段。郑洛新国家自主创新示范区获批开建。我市入选国家小微企业创业创新基地城市示范、电子功能材料区域集聚试点、循环经济示范城市。以空空导弹研究院、中航光电等为代表的先进国防科技工业取得长足发展，洛阳制造在航空航天等国家一系列重大工程中绽放异彩。

　　——开放合作开创新局面。现代开放体系加快构建，中国(河南)自由贸易试验区洛阳片区获批建设，牡丹文化节、河洛文化旅游节等平台作用进一步增强。全年签订亿元以上招商引资合同390个，投资总额1769.1亿元；已开工项目285个，其中10亿元以上项目36个。实现进出口总额17.1亿美元；其中与“一带一路”沿线国家进出口额7.84亿美元，占全市的45.8%。

　　——人民生活有了新提高。全年民生支出达378.4亿元，占全市一般公共预算支出的73.2%。67件民生实事中，除1件调整计划外，其它66件全部完成。脱贫攻坚初战告捷，214个贫困村46760名贫困人口如期脱贫。预计全市居民人均可支配收入达21120元，同比增长8%。

　　我们主要做了以下工作：

　　——坚持把稳增长保态势作为重中之重，综合施策、精准发力，经济运行稳中向好。将稳定即期增长与促进长远发展相结合，聚焦关键、破解难题，着力培育新的经济增长点。狠抓项目扩投资。396个省市重点项目完成投资1044亿元，占年计划的136.5%。新开工亿元以上项目649个，完成投资1739亿元。精细服务稳企业。建立地企合作双月联席会议制度，四次地企合作会议签约项目100个，总投资667.4亿元；签订金融对接协议47个、总金额2670.8亿元；50个产销对接合同金额达15.65亿元。开展“政策落实进万企”活动，实施“小巨人”企业培育和中小企业“百千万成长工程”，举办银企、用工、产销、产学研对接活动47场，为1515家企业提供还贷周转金61.9亿元。2次大型银企洽谈会签约资金826亿元，落实率90%以上。前11个月，全市规模以上工业利税、利润同比分别增长17.5%和24%，高于全省16.1和18个百分点。

　　——坚持把构建现代产业体系作为发展根基，强化创新驱动，深化结构调整，转型升级步伐加快。以“四双联动”为路径，以重大项目为支撑，促进动力转换、速度换挡、结构转型。全面推进科技创新。新增省级以上创新主体322家，其中高新技术企业38家、创新型试点企业8家。入选省创新型龙头企业、“互联网+”工业创新示范企业数量均居全省第一。新增市级以上研发平台211个，其中省重点实验室4个、工程技术研究中心17个。新增国家级科技企业孵化器4个、众创空间2个，省国际科技合作基地3个。荣获国家科技进步特等奖2项、技术发明奖1项，省重大科技专项6个。清华大学先进制造产业研发基地等一批新型研发机构落户洛阳。中国创新创业大赛先进制造行业总决赛、“直通硅谷”创业创新大赛成功举办。“河洛英才计划”新引进团队5个、人才87名。高新区入选国家科技服务业区域试点。中信重工成为国家首批企业“双创”示范基地、17个国家专业化众创空间之一，荣膺中国工业大奖、中国质量奖提名奖。全市万人有效发明专利7.6件，同比增长30%。推进产业优化升级。工业方面，实施中航光电新技术产业基地等重大结构调整项目339个，完成投资416亿元；中航锂电产业园三期等84个项目竣工投产。我市成为首个国家先进装备制造业标准化试点市。服务业方面，实施白云山自驾车营地等重大项目200个，完成投资376亿元；隋唐洛阳城九洲池、卫坡古村落文化旅游等43个项目竣工投用。全年接待游客1.14亿人次、旅游总收入905亿元，分别增长9.5%和16%。我市入选全国休闲农业乡村旅游示范市。栾川、嵩县、孟津、洛龙被授予国家全域旅游示范区创建单位。着力强化载体支撑。前11个月，全市产业集聚区完成投资1878.8亿元，占全市的46%。在全省观摩活动中，我市荣获小组第一；高新、偃师分别入选全省“十强”“十先进”产业集聚区，西工、洛龙分别入选全省“十强”“十先进”服务业“两区”。洛阳产城融合示范区获国家批准。

　　——坚持把推进新型城镇化作为战略任务，统筹做好“三篇文章”，城乡面貌持续改善。谋划实施“一中心六组团”城市发展战略，加强城乡规划、建设、管理，城市综合承载力进一步提升。增强基础支撑能力。龙门站综合交通枢纽、洛济高速(洛阳—济源)、尧栾西高速(尧山—栾川—西峡)等项目加快实施；洛吉快速通道黄河大桥、310国道新建、故县水库引水等工程完成过半；前坪水库成功截流；二广高速瀍河站互通式立交等工程竣工通车；三洋铁路、郑登洛(郑州—登封—洛阳)和焦济洛(焦作—济源—洛阳)城际铁路、大鱼沟抽水蓄能电站等项目前期工作取得新进展。华润首阳山电厂“引热入洛”工程全线贯通，全市新增供热面积364万平方米。110千伏伊滨区拓展等11项输变电工程建成投运。加大城市建设力度。海绵城市建设正式启动，城市地下综合管廊专项规划获批。城市轨道交通1号、2号线试验段和古城快速路等项目开工建设；孙辛路改造、玄奘路等一批市政道路竣工通车；环城路、城市生活垃圾综合处理园区、应天门遗址保护展示等工程顺利推进。棚户区改造完成征迁717.4万平方米、开工912.3万平方米、竣工520.3万平方米，货币化安置率达52.7%、高于全省19.5个百分点。河柴文化游园等47处游园建成投用。加大环境治理力度。实施蓝天、碧水等五大行动计划，扎实开展扬尘、机动车尾气、露天烧烤等专项治理，打好大气污染防治攻坚战。企业窑炉废气治理、发电机组超低排放改造、燃煤锅炉拆除及提标工作扎实推进。淘汰黄标车、老旧车2.4万辆。城区地表水断面、集中式水源地水质达标率保持在100%。造林40余万亩，抚育改造森林160万亩。绿色建筑数量、面积居全省首位。夯实农业农村基础。粮食总产46.3亿斤，属历史高产年份。新增国家出口食品农产品质量安全示范区2个，省级农业产业化集群3个、龙头企业7家，市级龙头企业47家。66个市级美丽乡村试点顺利推进，765个提升村、510个示范村建设任务全部完成。硬化通组通户道路2002公里。新增有效灌溉面积4.26万亩，治理水土流失面积153平方公里。扎实推进新型城镇化试点建设。出台居住证管理暂行办法和推进农业转移人口市民化工作方案，42.5万人在洛办理居住证登记。洛阳新型城镇化试点经验在全国推广。

　　——坚持以供给侧结构性改革为主线，突出问题导向，把握市场方向，发展活力进一步增强。4大类57项重点改革任务有序推进。供给侧结构性改革初见成效。扎实推进“三去一降一补”，关闭退出煤矿5处(6对)，化解过剩产能153万吨；商品房库存消化周期由2015年的23.5个月降为9个月；认真落实省、市降成本政策措施，减轻企业负担35亿元。国企改革步伐加快。33家驻洛央企“三供一业”分离移交工作稳步推进，6家驻洛省企分离企业办社会工作完成年度省定任务，首批23家市出资企业完成脱钩改制，混合所有制改革、国企内部“三项制度”改革等试点工作有序推进。“放管服”改革力度加大。深化行政审批、商事登记制度改革，在全省率先推行企业投资负面清单，全面取消非行政许可审批，调整、取消行政审批51项，审批环节、时限平均压缩20.9%和32.2%。投融资体制改革持续深化。启动实施“引金入洛”工程，平安银行、中原银行、中信建投、中原农业保险等金融机构顺利进驻，农商行组建工作全面完成。新增“新三板”挂牌企业11家，3家上市企业再融资11.3亿元。PPP模式试点工作扎实推进。同时，卫生、农林、交通、流通等领域改革持续深化，我市入选河南省内贸流通改革发展综合试点城市。

　　——坚持以改善民生为根本，强力推进民生工程，千方百计惠民利民，群众获得感持续增强。精准扶贫扎实推进。各级政府投入大量人力、财力、物力，建设易地扶贫搬迁安置点91个，完成整村推进项目461个，实施产业扶贫项目528个，贫困地区生产生活条件显著改善，基本公共服务水平进一步提高。社会保障能力持续加强。新增城镇就业13万人、农村劳动力转移就业3.1万人；发放创业担保贷款29.8亿元，扶持2.3万人创业，带动8.1万人实现就业。国家大学科技园、恒生科技园入选全国创业孵化示范基地。城乡居民医疗保险待遇和城乡低保、农村五保供养标准进一步提高。公共服务不断完善。新建、改扩建公办幼儿园48所，入园率高于全省4.5个百分点；城市区义务教育全面消除超大班额；普通高招再创新高。改扩建县级医院1所、乡镇卫生院9所。建成社区养老服务中心30个，新建农村老年幸福院202个，新增养老床位3400张。建成基层综合文化服务中心10个，举办“舞台艺术送农民”等公益性演出700余场，开展群众性文化活动2000余次。市体育中心、洛浦公园全民健身提升等工程竣工投用，乡、村两级体育健身设施覆盖率达100%。我市成功创建国家公共文化服务体系示范区，入选首批国家文化消费试点城市，顺利通过省全民健身示范市验收。社会大局和谐稳定。积极回应群众合理诉求，荣膺“人民网网民留言办理工作10周年贡献奖”。持续开展安全生产大检查，加强重点行业领域专项整治，安全形势总体平稳。加强食品药品市场监管，保障群众饮食用药安全。严惩各种违法犯罪，社会治安形势良好。

　　——坚持以“两学一做”学习教育为载体，致力勤政廉政，改进政风行风，政府效能不断提高。深入开展“两学一做”学习教育。着力强化“四个意识”，认真履行全面从严治党主体责任，有的放矢整改问题，政府系统党员干部作风进一步好转。加强法治政府建设。自觉接受人大及其常委会监督，积极支持政协参政议政，办理人大代表建议296件、政协提案498件。完善重大行政决策制度，推进依法科学民主决策。建立政府法律顾问制度，组建了市政府法律顾问团。审查办理地方性法规草案2部、规章2件。我市在2016年中国法治政府评估中名列全省第一。强力推进政府廉政建设。公车改革取得阶段性成果。“三公经费”同比下降34.1%。坚持以零容忍态度惩治腐败，政治生态不断净化。军政军民团结进一步加强，民兵预备役工作取得新进步，我市荣膺全国双拥模范城“七连冠”。同时，民族宗教、外事侨务、对台、统计、审计、人防、气象、地震、史志、残疾人工作，妇女儿童事业等均取得了新的成绩。

　　各位代表！以上成绩的取得，离不开省委、省政府和市委的坚强领导，离不开历届政府班子和老同志打下的坚实基础，离不开市人大、市政协、各民主党派、人民团体的监督支持，更离不开全市人民和社会各界的团结奋斗、无私奉献。在此，我代表市人民政府，向辛勤工作在各条战线上的700万洛阳人民，向人大代表、政协委员，向各民主党派、人民团体，向离退休老领导、老同志，向驻洛部队、武警官兵和公安干警，向所有关心支持洛阳发展的社会各界和海内外朋友，表示衷心的感谢并致以崇高的敬意！

　　肯定成绩的同时，我们也清醒地看到，全市经济社会发展中还存在不少问题：实体经济活力不足，经济下行压力较大；战略性新兴产业、现代服务业和高效农业占比偏低，产业转型升级任务艰巨；科技投入不足，产学研结合度较低，科技成果转化能力不强；改革攻坚进入深水区，国企改革任务繁重，深层次矛盾需要破解；对外开放程度不够，开放载体和平台不足；公共服务体系不完善，就业、就医、教育、住房、环保等问题亟待进一步解决；政府职能转变不到位，发展环境尚需进一步优化。对于这些问题，我们决不推诿、决不回避，一定尽全力予以解决。

　　二、2017年总体要求及重点工作安排

　　2017年是贯彻落实省、市党代会精神，实现洛阳新定位的开局之年，也是供给侧结构性改革的深化之年，洛阳既迎来了难得的发展机遇，又面临着诸多的困难和挑战。

　　新定位赋予了洛阳光荣艰巨的历史使命，我们要勇于担当、奋发有为，为中原更加出彩提供坚实有力的支撑！习近平总书记要求河南打好“四张牌”；国家《促进中部地区崛起“十三五”规划》提出，要继续做大做强洛阳等区域性中心城市；省十次党代会强调，要“巩固提升洛阳中原城市群副中心城市地位，建设全国重要的现代装备制造业基地和国际文化旅游名城，推动豫西北各市与洛阳联动发展，形成带动全省经济发展新的增长极”，为洛阳加快发展指明了方向、增添了动力。只要我们牢记使命、久久为功，就一定不会辜负党中央、国务院和省委、省政府的重托！

　　新机遇鼓舞了洛阳人民率先发展、科学发展的信心，我们要乘势而上、厚植优势，为洛阳赶超跨越积蓄更加强劲的能量！国家“一带一路”倡议深入推进，郑洛新国家自主创新示范区、中国(河南)自由贸易试验区相继获批，战略组合叠加效应逐步显现，为洛阳加快发展创造了良好的政策环境。只要我们抓住机遇、乘势而上，就一定能够在新一轮城市竞争中占据主动、拔得头筹！

　　新目标描绘了洛阳未来的宏伟蓝图，我们要牢记使命、砥砺前行，全面开启率先发展的新征程！市十一次党代会确立了“9+2”工作布局和“四高一强一率先”的奋斗目标，描绘了“十三五”洛阳经济社会发展的宏伟蓝图。只要我们紧盯目标不动摇、咬定青山不放松，凝心聚力、务实重干，就一定能把蓝图变为现实，谱写决胜全面小康、让中原更加出彩的洛阳篇章！

　　新形势带来了更多的风险挑战，我们要迎难而上、负重奋进，全力开创转型发展的新局面！当前，外部环境依然复杂多变，新常态下经济发展的不确定性明显增加；区域竞争日趋激烈，新的发展格局正在形成；自身发展的深层次矛盾尚未有效破解，产业升级、经济转型倒逼压力增大。但是，只要我们迎难而上、奋力拼搏，就一定能够实现转型发展、跨越发展、率先发展！

　　根据市委全会和市委经济工作会议精神，今年政府工作的总体要求是：深入贯彻习近平总书记系列重要讲话精神，深入贯彻党中央治国理政新理念新思想新战略，认真落实中央、省委、市委经济工作会议，省、市党代会，省委、市委全会精神，统筹推进“五位一体”总体布局和协调推进“四个全面”战略布局，坚持以新发展理念为指导、以提高经济发展质量和效益为中心、以推进供给侧结构性改革为主线，坚持稳中求进工作总基调、稳中求新、稳中求好，坚持发挥优势打好“四张牌”，坚持以体系为支撑、以专项为带动、以项目为抓手，坚持以人民为中心的发展思想，全面做好稳增长、促改革、调结构、惠民生、防风险各项工作，加快推进“9+2”工作布局、实现“四高一强一率先”奋斗目标，以优异成绩迎接党的十九大胜利召开。

　　今年全市经济社会发展的主要预期目标是：地区生产总值增长8.8%、在实际工作中力争更好一些，一般公共预算收入增长8.5%，全社会固定资产投资增长13%，社会消费品零售总额增长12%，进出口总值保持稳定，城乡居民收入与经济增长基本同步，居民消费价格涨幅控制在3%左右，城镇新增就业10万人以上，常住人口城镇化率提高1.65个百分点，节能减排、环境保护等约束性指标完成省下达任务，发展质量和效益明显提高。

　　提出这样的预期目标，既保持了全市经济发展速度的连续性，又明显高于全省发展目标，与中央、省要求和洛阳市“十三五”发展目标相一致，与洛阳全国区域性中心城市、中原城市群副中心城市的发展定位相适应，与实现“四高一强一率先”的奋斗目标相吻合，在实际工作中，我们要争取更好的结果。

　　重点抓好以下十个方面的工作：

　　(一)稳定经济运行，壮大综合实力，努力巩固稳中向好的发展势头。坚持把稳定经济增长作为经济工作的突出任务，以稳保进、以进固稳，稳中求新、稳中求好。一是精细服务稳企业。实施企业“倍增提质”计划，加强运行监测，搞好要素保障，坚持分类施策，创新服务机制，不断提高质量效益。提升轨道车辆、成套装备、铁路轴承、光电连接器等优势产品的市场份额，支持光伏企业拓展应用市场，推动有色金属行业调整改造提升。抢抓新一轮国家电力体制改革机遇，积极开展大用户直购电交易。办好银企洽谈会，全年计划新增贷款320亿元以上。二是狠抓项目增投资。大力实施“9643”合理有效投资行动计划，突出抓好415个总投资3295亿元的省市重点项目。全年计划实施亿元以上项目1400个，完成投资2800亿元，其中新开工项目400个以上，推动一批重大项目前期工作取得突破。抓住国家有序放开配电网、电信、市政等领域市场准入的机遇，鼓励引导民间资本进入。三是多策并举促消费。落实国家“十大扩消费行动”，着力扩大住房、旅游、文化等领域消费，积极培育健康养老、体育健身等新型消费业态，增强区域消费辐射效应。

　　(二)突出示范带动，优化创新生态，加快构建现代创新体系。大力推进创新型城市建设，加快实施8个重大专项，争当郑洛新国家自主创新示范区的领头羊、全省创新发展的排头兵。一是加快建设郑洛新国家自主创新示范区。科学编制洛阳片区发展规划，围绕开放协同创新、科技成果转化、科技金融结合、人才培养引进等方面先行先试，完善政策支持体系，进一步激发创新活力。二是推动大众创业万众创新。以国家小微企业创业创新基地城市示范为载体，依托中信重工、高新区等国家、省级“双创”示范基地，大力实施“重微共振”工程，建设13个小微企业公共服务平台、提升20个创新创业载体，积极争取国家第二批“双创”示范基地，打造最具活力的国家创业创新示范城市。三是深化科技体制改革。将高校、科研院所专业技术人员科技成果转化情况纳入绩效评价体系，激发广大科研人员的积极性、创造性。依托科技大市场网上交易服务平台和线下服务大厅，吸引、集聚科技资源，建设豫西北乃至全省科技资源交易中心。四是强化创新平台支撑。大力推进创新主体、创新平台“双倍增”行动，实施创新龙头企业和“科技小巨人”企业培育工程，重点培育400家创新主体，新增研发平台260家。支持洛阳中科信息产业研究院等加快建设；围绕装备制造、新能源、新材料等领域，建设提升20个产业技术创新战略联盟；推动骨干企业、科研院所建设一批新型研发机构。支持有条件的县(市)建设科技园区和孵化器，提升县域创新发展能力。五是优化创新生态体系。引导金融资金、社会资本进入技术创新领域，探索设立科技创业种子投资引导资金、天使投资引导资金、科技成果转化风险补偿资金，满足科技型企业融资需求。推进知识产权质押融资，鼓励科技型企业上市融资或挂牌交易。大力实施“河洛英才计划”“河洛工匠计划”，培养引进一批具有重大技术突破和较强产业化能力的领军型、紧缺型人才团队。实施标准引领、质量认证和品牌带动工程，提升全市质量水平。

　　(三)强化项目支撑，振兴实体经济，加快构建现代产业体系。围绕“565”现代产业体系，大力实施8个重大专项，加快新旧动能转换，积极创建“中国制造2025”试点城市、国家老工业城市转型升级示范区，促进产业向高端化、集群化发展。一是做大做强五大主导产业。计划实施重大项目337个，完成投资616亿元。重点推进中信重工节能环保装备产业化基地、中原大数据云计算中心等197个续建项目；争取洛阳石化炼油扩能改造“一主四辅”和煤制氢项目、重渡沟国家旅游度假区等140个项目开工建设。二是积极培育六大新兴产业。计划实施重大项目160个，完成投资351亿元。重点推进中国一拖新型轮拖智能制造工厂、民生药业集团二期等97个续建项目；争取中航光电智能机器人产业化等63个项目开工建设；国舰机器人核心部件产业化、格力电器物流配送中心二期等项目竣工投产。三是大力发展五大特色产业。计划实施重大项目179个，完成投资327亿元。重点推进龙门石窟世界文化遗产园区旅游商业设施等125个续建项目；争取河科大二附院综合楼等54个项目开工建设；科大讯飞洛阳语音云创新研究院等项目建成投用。四是提高融合发展水平。围绕航空航天、后勤保障、装备制造、新材料等领域，谋划实施一批军民两用战略性新兴产业重点工程，组建军民融合创新研究院，争创国家军民融合创新示范区，加快建设国家级军民结合产业示范基地。大力发展制造业服务业，促进先进制造业与现代服务业融合发展。积极创建产业与金融合作试点城市，加快建设文化和科技融合示范基地。五是推动产业集聚发展。大力实施主导产业升级、创新能力提升、服务功能完善、产城融合发展等行动计划，抓好集聚区体制机制创新、产业布局优化、闲置土地利用等工作，推动产业集聚区提质转型。全市产业集聚区计划新开工亿元以上主导产业项目165个，竣工120个。启动服务业专业园区规划建设，推动商务中心区和特色商业区提质增效，实施产业项目90个，完成投资190亿元。

　　(四)聚焦改革攻坚，激发市场活力，加快构建现代市场体系。全面深化各项改革，实施4个重大专项，让改革红利加速释放、市场活力充分迸发！一是深化供给侧结构性改革。围绕去产能，严控水泥、平板玻璃等行业新增产能，以法治化、市场化手段淘汰落后产能，防止已淘汰产能死灰复燃；大力实施新一轮技术改造，支持企业采用新技术、新产品、新设备提质增效。围绕去库存，落实好信贷、税收优惠政策，加大棚户区改造力度，鼓励农村和外来人员进城购房，推动房地产业持续健康发展；大力发展住房租赁市场，推进租购并举，满足刚性需求，化解商品房库存；对库存量较大的县(市)区，减缓用地供应，加大货币化安置力度。围绕去杠杆，把降低企业特别是国有企业的杠杆率作为重中之重，盘活企业存量资产，引导企业通过债转股、股权融资、挂牌上市、股本增发、再融资等方法降低负债率；抓好挂牌上市后备企业梯队培育，提高直接融资比重。围绕降成本，重点在减税、降费、降低要素成本上下功夫，深入推进“放管服”改革，深化涉企收费专项整治，进一步降低制度性交易成本，减轻实体经济负担；以减少“跑冒滴漏”为着力点，引导企业“眼睛向内”降本增效。围绕补短板，紧盯制约经济社会发展的重要领域、关键环节和人民群众急需解决的突出问题，加大对基础设施、民生保障、公共服务、脱贫攻坚、环境保护等方面的投入力度，进一步优化财政支出和投资结构，增强发展的协调性和可持续性。二是深化国资国企改革。支持中钢洛耐、中铝洛铜等央企深化改革、转型升级，扎实推进驻洛央企“三供一业”分离移交；推动洛铜集团、LYC轴承等省企开展混合所有制改革，全面完成剥离企业办社会职能；分类推进市(县)属企业深化产权制度改革，全面完成市出资企业脱钩改制，推动有色矿业、万基控股等企业改革取得突破性进展。持续深化地企合作，扩大合作成果。三是大力推进农业供给侧结构性改革。积极实施高标准粮田“百千万”建设工程、特色农业“百乡百村”示范创建工程、新型农业经营主体“百社百场”示范培育工程，建设高标准粮田4万亩，新增市级以上示范社50家以上、示范家庭农场30家以上，提高农产品质量和效益。实施特色沟域经济重大专项，建成10条沟域经济产业带。抓好农村产权制度改革、供销系统综合改革，扎实开展扶持村级集体经济和农村集体经济股份合作试点，稳步推进水利、集体林权和国有林场等各项改革，完成土地确权登记颁证工作。四是深化金融体制改革。持续实施“引金入洛”工程，引进恒丰银行、广发银行等金融机构，加快洛阳银行上市步伐，壮大地方金融主体。推进15家企业上市(挂牌)、再融资，实现资本市场融资185亿元以上。积极推广PPP模式，扩大融资成果。鼓励支持金融创新，引导设立各类创业投资、股权投资和并购基金，探索发行小微企业增信集合债、集合票据、双创债，不断丰富金融业态。五是深化其他领域改革。按时完成城市综合执法体制改革，统筹推进财政、教育、文化、价格、交通、公务用车等领域改革。六是加快发展非公经济。积极培育国家、省级中小企业公共服务平台、小微企业创业创新示范基地。设立产业发展基金，拓宽民营企业融资渠道。鼓励民营企业参与混合所有制改革、与科研院所建立合作研发和成果转化机制。继续实施“小巨人”企业培育工程，重点建设钨钼铝钛材料精深加工等10个小微企业工业园。七是完善现代市场体系。加强消费基础设施建设，改造提升中心城区大型市场，做强市场功能，做优市场环境，汇聚更多的人流、物流、信息流、资金流。加快推进国家电子商务示范城市建设，提升农村电商发展水平。

　　(五)完善载体功能，优化空间布局，加快构建现代城镇体系。科学推进新型城镇化，实施6个重大专项，加快形成“四级联动”城镇总体布局。一是强力推进“一中心六组团”城市发展战略。完成四期总规实施评估，启动五期总规修编，高标准编制“一中心六组团”战略规划，推动中心城区与六组团产业承接、交通连接、生态对接，打造“半小时经济圈”。“一中心六组团”计划开工项目268个、竣工135个，完成投资561.6亿元。二是优化中心城区功能布局。加快地下综合管廊、海绵城市试点建设，大力推进“一环线、12座立交”等一批重点市政工程。洛北城区要以疏解提升为主，启动建设王城大道快速化、洛吉快速通道与定鼎路互通立交等项目，确保一季度河洛路改扩建工程开工，牡丹文化节前新街跨洛河大桥竣工、新伊大道开工，上半年九都路东延长线工程竣工。洛南城区要着力完善公共服务功能，确保东环路二期、南环路牡丹文化节前完工，古城快速路一期项目上半年完工、二期项目4月开工；开工建设龙门站综合交通枢纽配套项目，加快动车停车场前期工作，确保与城际铁路同步开建。伊滨城区要加快核心区建设，拉开框架，尽快实现与周边城区连接，重点抓好汉魏大道等主干路网建设，力争科技大道等项目牡丹文化节前建成通车，伊滨路与顾龙路7月实现连通。三是加快新型城镇化进程。全面启动洛阳国家产城融合示范区建设，高质量完成国家新型城镇化综合试点建设任务，确保常住人口城镇化率达到55.8%以上。积极争取国家城乡交通一体化试点，开通“六组团”城乡公交车。棚户区改造完成征迁680万平方米，新开工安置房5.62万套，基本建成3.5万套。抓好偃师、孟津、新安“百城建设提质示范工程”，实施县城管理三年提升行动计划，创建一批全国文明县城、国家园林县城和卫生县城。实施特色小镇培育工程，打造一批工业强镇、商贸重镇、旅游名镇。四是提升城市管理水平。大力推进城市修补、生态修复“双修”示范城市建设，开展广告整治、绿化提升、亮化改造和山体、水系、绿地修复。抓好文明城市创建工作，改造提升背街小巷，确保90%以上市区主干道实现机械化清扫。有序推进数字化城市管理系统二期工程建设，实现城市管理平台配网运行、生活垃圾综合处理园区试运行。五是加快建设美丽乡村。扎实推进农村人居环境提升村和示范村创建活动，确保27%以上的行政村达到提升村创建标准，18%以上的行政村达到示范村创建标准。持续抓好农村秸秆禁烧。

　　(六)强化辐射带动，提升承载能力，加快构建现代基础设施体系。充分发挥基础设施对经济社会发展的支撑引领作用，大力实施8个重大专项，推动基础设施协调共建、均衡发展。一是完善综合交通体系。围绕建设洛阳全国性枢纽，做好洛阳机场改扩建三期、焦济洛城际铁路、栾卢高速(栾川—卢氏)、渑栾高速(渑池—栾川)等项目前期工作；积极推进尧栾西高速、洛济高速等项目建设；确保二广高速市区段扩宽改造工程上半年开工，新汝高速(新安—宜阳—伊川—汝阳)下半年开工，力争郑登洛城际铁路年底前开工。加快建设4A级以上景区快速通道和县乡之间二级公路，确保洛吉快速通道黄河大桥10月主体建成、12条干线公路年底前建成通车。抓好城市快速路和快速公交“双快系统”建设，对环城高速公路重点出入口进行提升改造，确保城市轨道交通1号线上半年开工、力争2号线下半年开工。二是提升综合承载能力。持续实施城镇基础设施扩能增效工程，加快东环线燃气管网和伊川至嵩县、栾川天然气管道建设，抓好500千伏洛阳东等43项电网工程；积极推进神华国华孟津电厂等“引热入洛”工程，新增供热面积800万平方米，集中供热普及率达60%。三是提高农业综合保障能力。抓好中小河流治理、饮水安全巩固提升，完成小浪底南岸灌区前期工作、前坪水库年度建设任务、故县水库引水工程，新增有效灌溉面积3万亩，治理水土流失面积150平方公里。完成60个小城镇(中心村)、100个贫困村电网改造。建设农村公路600公里、桥梁2800延米。

　　(七)传承历史文脉，彰显文化特色，加快构建文化传承创新体系。加强历史文化保护、传承和利用，实施5个重大专项，着力打造国际文化旅游名城和华夏历史文明传承创新示范区。一是彰显历史文化名城底蕴。编制完成全市域文物保护与利用总体规划，大力推进隋唐洛阳城应天门、定鼎门片区、南城墙遗址公园和汉魏故城太极殿、汉函谷关等一批遗址保护展示工程，加快建设全国大遗址保护示范基地。积极推进二里头夏都遗址博物馆、丝绸之路博物馆、仓窖博物馆、古代壁画保护研究中心建设，支持民办博物馆发展。扎实推进老城历史文化街区、涧西工业遗产群和传统村落保护整治。加强非物质文化遗产传承展示保护。二是做大做强文化旅游产业。以“旅游+”和“全域旅游”理念为引领，加快智慧旅游试点城市建设和4个全域旅游示范区创建，推动文化、旅游深度融合。重点推进灵山文化旅游园区等一批文化旅游项目。大力开展国家、省级旅游度假区、A级景区创建，重点打造以老君山、白云山等为代表的生态休闲度假游品牌，以精品化景区带动旅游业提档升级。积极发展农耕文化、都市农业等乡村旅游，让群众望得见山、看得见水、记得住乡愁！加快国家文化消费试点城市建设，大力发展滑雪、漂流、汽车营地等体育旅游产品，温泉养生、医疗养老等康体旅游产品，实景演艺、影视演出等文化旅游产品，形成多元化的旅游产品体系。办好牡丹文化节、河洛文化旅游节、市十三届运动会暨全民健身大会等重大节会。全年接待游客1.23亿人次，实现旅游总收入1040亿元。三是完善公共文化服务体系。巩固国家公共文化服务体系示范区创建成果，实施图书馆、文化馆提升改造工程，规划建设科技馆新馆、方志馆，确保城市规划馆建成投用，争取建成市文化馆、少儿图书馆、非遗展示中心。大力开展“百场公益性文化演出”等文化惠民活动，创作一批具有洛阳特色的优秀艺术作品。

　　(八)加强环境保护，狠抓污染治理，加快构建生态环境建设体系。牢固树立绿色发展理念，大力实施5个重大专项，统筹推进“五大行动计划”，努力建设生态宜居的美丽家园。一是加强环境综合整治。持续打好大气污染防治攻坚战，狠抓燃煤、工业、扬尘、油品、黄标车等重点领域污染治理，加快推进煤改电、煤改气，集中整治“小散乱差”污染企业，彻底消除污染围城问题。全面打响水污染治理攻坚战，认真落实“河长制”，加快水生态文明试点市建设，大力推进“四河同治”，加强河渠截污、黑臭水体治理和污水管网建设，完成县城黑臭水体垃圾清理、截污纳管。加强城镇污水、污泥处理设施建设与提标改造，污水处理厂全部执行一级A排放标准，污泥无害化处理处置率达90%以上。二是构筑绿色生态屏障。实施廊道绿化、城郊森林提升、荒山绿化工程，建设环城沿路生态防护隔离带，完成廊道绿化提升1045公里、5万亩；对周山等城市森林公园实施改造提升，完善休闲娱乐、体育健身等服务功能；加强天然林保护、湿地公园建设。实施51项园林绿化工程，加快洛浦公园提升改造，持续抓好中心城区道路、公园、园林绿化提升及小游园建设，扩大城市绿量。三是大力发展循环经济。加强矿产资源及尾矿资源综合利用，推进建筑垃圾、生活垃圾与污水处理厂污泥循环利用，完成餐厨废弃物资源化利用和无害化处理试点建设。实施产业集聚区、专业园区循环化改造工程，重点推进中再生洛阳公司报废汽车拆解等项目，确保全市资源产出率同比提高5%以上。

　　(九)加强平台建设，扩大双向开放，加快构建现代开放体系。以中国(河南)自由贸易试验区建设为契机，大力实施5个重大专项，全方位融入“一带一路”倡议，打造中西部对外开放新高地。一是加快开放平台建设。认真编制中国(河南)自由贸易试验区洛阳片区实施方案和产业发展、空间布局等规划，扎实开展各项改革试点，吸引高端人才集聚和知名企业落户。抓好综合保税区申建，完成空港经济区规划编制，支持建设中储保税物流中心。积极推进铁路口岸和铁路物流园区场站建设，争取开通铁路国际货运班列。完善洛阳航空口岸功能，提升机场航空货运保障能力。力争创建省级以上出口食品农产品质量安全示范区2个以上。二是大力推进精准招商。发挥科研院所和产业基础优势，围绕“565”现代产业体系和60个重大专项，开展“一对一”产业专题对接活动，积极承接发达地区链条式、集群式产业转移，吸引龙头企业、关联企业入驻，引进10亿元以上重大项目不少于80个。三是推动外贸稳定增长。大力培育、引进“两头在外”企业，加快建设跨境电子商务综合实验区，积极培育外贸综合性服务企业、服务外包示范园区，力争洛阳外贸公共服务平台建成运营。四是加快企业“走出去”步伐。支持优势企业到境外投资，参与国家级合作工业园开发，开拓“一带一路”沿线新兴市场。推动对外承包工程与外派劳务有机结合，鼓励中铁隧道集团、石化工程公司等对外承包工程，带动技术、标准和大型机械及成套设备出口。

　　(十)倾力改善民生，增进群众福祉，加快构建公共服务体系。坚持以人民为中心的发展思想，持续加大民生投入，积极实施5个重大专项，办好省定、市定民生实事，让洛阳人民的日子一年更比一年好！一是坚决打赢脱贫攻坚战。扎实推进精准扶贫、精准脱贫，大力实施“五个一批”，抓好产业扶贫、金融扶贫、易地扶贫搬迁等重大项目建设，完成198个贫困村、8.12万贫困人口脱贫任务，确保伊川脱贫摘帽，偃师、洛龙、伊滨整体脱贫。二是稳定就业促进创业。大力实施全民技能振兴工程，持续推进创业孵化基地、创业园区建设，完善就业创业服务体系，发放创业担保贷款15亿元，以创业带动就业。创新“互联网+高校毕业生服务”，实现高校毕业生高质量就业。鼓励农民工返乡创业，完成创业培训1万人、农村劳动力技能就业计划3万人。新增城镇就业10万人、农村劳动力转移就业2.6万人。三是努力办好人民满意的教育。启动第三期学前三年行动计划，新建、改扩建公办幼儿园63个，扩大公办、普惠性幼儿园覆盖率。加快实施义务教育“全面改薄”、城镇义务教育资源扩充五年计划，新建、改扩建农村中小学151个，推动义务教育均衡优质发展。继续做好高中阶段教育普及工作，不断提高教学质量。高质量完成职业教育资源整合，加快推进职业技术学院、幼儿师范学校搬迁改造及职教园区建设；支持驻洛高校改革发展，促进高校一流学科建设。鼓励、规范民办教育发展。四是织密织牢社会保障网。扎实推进机关事业单位养老保险制度改革。抓好城镇居民大病保险试点、城镇居民基本医疗保险和新农合制度整合工作。加强征缴管理，确保各项社保资金按时足额发放。实施好“菜篮子”工程，让人民群众吃上“放心菜”“便宜菜”。加强社会救助体系建设，切实保障困难群众基本生活。积极推进国家养老服务业综合改革和医养结合试点城市建设，整顿改造提升现有养老机构，提高农村敬老院集中供养水平，新建养老床位3000张。加大保障性住房建设力度，基本建成公共租赁房5000套，分配入住4000套。改造农村危房5000户。五是着力打造健康洛阳。坚持把人民健康放在优先发展的战略地位，推进分级诊疗制度建设，完善社区卫生服务网络，提高公共医疗服务水平，打造“15分钟就医圈”。积极推动洛阳正骨国家中医骨伤区域医疗中心建设。落实全面两孩政策，促进人口均衡发展。加快县(市)“两场三馆”体育设施建设，打造“15分钟健身圈”。六是切实维护社会稳定。落实安全生产责任，加强隐患排查治理，健全事故防控体系，实施尾矿库标本兼治工程，坚决防范各类生产安全事故发生。加强食品安全监管，切实守住舌尖上的安全。持续推进平安洛阳建设，严厉打击违法犯罪活动。做好新形势下的群众工作，加强矛盾排查化解，有效防范和打击非法集资，确保社会和谐稳定。积极支持国防和军队改革，推动军民融合发展，提升新形势下国防动员、国防教育、设施保护、双拥等工作质量。继续做好民族宗教、外事侨务、对台、统计、审计、人防、气象、地震、档案、残疾人工作、妇女儿童事业等工作。

　　三、加快建设人民满意的服务型政府

　　持续加强和改进政府工作，规范行政权力运行，推动作风建设常态长效，不断提高政府执行力、公信力和服务水平，树立为民务实清廉的政府形象。

　　(一)深化简政放权，加快转变政府职能。大力推进行政审批标准化试点建设，持续深化行政审批制度改革，严格控制新设行政许可，大力推行权力清单、责任清单、负面清单制度并实行动态管理，清理规范行政审批中介服务。全面推进“两集中两到位”职能归并改革，深化商事制度改革，进一步优化审批流程，努力形成更具吸引力的法治化、便利化营商环境。

　　(二)推进依法行政，切实维护公平正义。坚持法定职责必须为、法无授权不可为，建立健全市、县、乡三级政府法律顾问网络，大力推进服务型行政执法建设，依法设定权力、行使权力、制约权力、监督权力。严格执行人大及其常委会决定，提高人大代表议案、建议和政协提案办理质量，自觉接受人大及其常委会法律、工作监督和政协民主监督，认真听取民主党派、工商联、无党派人士和各人民团体意见，高度重视社会监督和舆论监督。大力推进政务公开，加强政策解读，回应社会关切，确保权力在阳光下运行。加强信用体系建设，着力打造诚信洛阳。健全科学民主决策机制，完善公众参与、专家论证、风险评估、合法性审查、集体讨论决定等重大行政决策程序，使政府的每一项决策都遵从法律法规、体现社情民意、彰显公平正义！

　　(三)坚持抓实抓长，全面提升效能效率。加快智慧城市和国家信息惠民试点城市建设，大力推进“互联网+政务服务”，加快市民中心建设，高标准打造贯通市县的政务服务平台，做到政务服务事项“应上尽上、全程在线”，让信息多跑路、让群众少跑腿、让企业更满意！大力倡树“清新简约、务本责实”作风，提升公务人员素质，优化政府工作流程，推动政府工作提速、提质、提效。

　　(四)从严正风反腐，着力打造廉洁政府。牢固树立“四个意识”，落实全面从严治党主体责任和监督责任，认真贯彻中央八项规定精神，严格遵守政治纪律、政治规矩，深入开展政风行风建设，驰而不息纠正“四风”，严肃查处懒政怠政为官不为行为。强化行政监察和审计监督，对公共资金、国有资产、国有资源和领导干部履行经济责任情况实现审计全覆盖。扎实开展廉洁教育和廉洁文化建设，从源头上遏制和预防腐败行为。注重家风传承，以良好的家风促政风带民风。

　　各位代表！新定位带来新机遇，新目标开启新征程。让我们紧密团结在以习近平同志为核心的党中央周围，在省委、省政府和市委的坚强领导下，团结奋进、积极作为，为早日实现“四高一强一率先”奋斗目标，谱写决胜全面小康、让中原更加出彩的洛阳篇章而努力奋斗！
